# Spearing for Timber

**Published:** 1883-08

**Authors:** 


### SPEARING FOR TIMBER.

A new industry has recently been developed in Ireland—a sort of timber prospecting never dreamed of by our American pine hunters. It is a well-known geological fact, says the *Northwestern Lumberman*, that immense tracts what are now bog lands in Ireland were once covered with forests of oak and pine, and that in cutting peat, immense trees of these varieties are found imbedded in the earth at depths of ten, twenty and thirty feet, in many cases whole groves being found standing just as they grew. To find out the location of these miniature subterranean forests is now the speculative work in which some industrious Irishmen are engaged. The timber, when brought to the surface, is found to be perfectly sound, and the oak, which is as black as ebony, is used extensively for ornaments of jewelry and fancy cabinet work, and sells at high prices. A recent visitor to the wild moor and mountain region of Donegal thus describes the way in which the seekers after buried forests operate: Two men, armed with steel rods about thirty feet long, traverse the bog, and by running their rods into the ground are able to ascertain where the trees are to be found. They work by what may be termed natural mathematics, and quickly determine the length of their prize, its approximate diameter, whether it is pine or oak, and is or is not a clumper—one of a company or clump. They fix on twenty or thirty feet square, and cross it with their searchers, say north and south, and then east and west, search it across each way, a stab to each foot or so, and in the course of a few minutes they know whether that area contains what they are looking for. The square lying next and next, and all near each other, are so searched, and the discoveries, if any, marked for future action. The unproductive are also marked, to avoid future loss of labor.

